# Anti-MPER antibodies with heterogeneous neutralization capacity are detectable in most untreated HIV-1 infected individuals

**DOI:** 10.1186/1742-4690-11-44

**Published:** 2014-06-07

**Authors:** Luis M Molinos-Albert, Jorge Carrillo, Marta Curriu, Maria L Rodriguez de la Concepción, Silvia Marfil, Elisabet García, Bonaventura Clotet, Julià Blanco

**Affiliations:** 1 IrsiCaixa-HIVACAT, Institut de Recerca en Ciències de la Salut Germans Trias i Pujol (IGTP), Hospital Germans Trias i Pujol, UAB, Badalona, 08916 Barcelona, Catalonia, Spain; 2 UVic-UCC, 08500 Barcelona, Spain

## Abstract

**Background:**

The MPER region of the HIV-1 envelope glycoprotein gp41 is targeted by broadly neutralizing antibodies. However, the localization of this epitope in a hydrophobic environment seems to hamper the elicitation of these antibodies in HIV infected individuals.

We have quantified and characterized anti-MPER antibodies by ELISA and by flow cytometry using a collection of mini gp41-derived proteins expressed on the surface of 293T cells. Longitudinal plasma samples from 35 HIV-1 infected individuals were assayed for MPER recognition and MPER-dependent neutralizing capacity using HIV-2 viruses engrafted with HIV-1 MPER sequences.

**Results:**

Miniproteins devoid of the cysteine loop of gp41 exposed the MPER on 293T cell membrane. Anti-MPER antibodies were identified in most individuals and were stable when analyzed in longitudinal samples. The magnitude of the responses was strongly correlated with the global response to the HIV-1 envelope glycoprotein, suggesting no specific limitation for anti-MPER antibodies. Peptide mapping showed poor recognition of the C-terminal MPER moiety and a wide presence of antibodies against the 2F5 epitope. However, antibody titers failed to correlate with 2F5-blocking activity and, more importantly, with the specific neutralization of HIV-2 chimeric viruses bearing the HIV-1 MPER sequence; suggesting a strong functional heterogeneity in anti-MPER humoral responses.

**Conclusions:**

Anti-MPER antibodies can be detected in the vast majority of HIV-1 infected individuals and are generated in the context of the global anti-Env response. However, the neutralizing capacity is heterogeneous suggesting that eliciting neutralizing anti-MPER antibodies by immunization might require refinement of immunogens to skip nonneutralizing responses.

## Background

The highly conserved Membrane Proximal External Region (MPER) of the gp41 HIV-1 glycoprotein contains linear epitopes targeted by the broadly neutralizing antibodies (bnAbs) 2F5, 4E10 and 10E8; all isolated from HIV-1 infected subjects [1-4]. The ability of the human immune system to mount a neutralizing response against this region and their protective activity in animal models [5] made the MPER a promising target for vaccine design aiming to develop a protective neutralizing response against HIV-1 [6-8]. However, the elicitation of such neutralizing responses against the MPER is challenging likely because of its poor immunogenicity due to topological constraints or to the existence of immunodominant nonneutralizing regions within gp41 [7,9,10]. Furthermore, some of the features presented by both 2F5 and 4E10 antibodies including lipid recognition and autoreactivity, represent a considerable immunological barrier when designing immunogens aiming to mimic anti-MPER responses [11-13]. The development of the B-cell cloning technology led to the recent isolation of the monoclonal antibody 10E8 [4], which is among the broadest and most potent neutralizing antibodies identified to date. Although it was shown initially to lack the limiting features presented by the previous anti-MPER bnAbs [4,14], it has been shown that 10E8 does bind membrane lipids by two hydrophobic residues in the CDRH3 loop, suggesting that anti-MPER bnAbs could mediate neutralization by similar mechanisms where the binding to the viral membrane plays a role [15]. Despite this controversy, it seems that the presentation of MPER epitopes in a lipid environment or in a soluble form may modify its recognition by anti-MPER antibodies [16,17].

The efforts to characterize bnAbs against the MPER have abridged the full characterization of other anti-MPER humoral responses, which also include several antibodies with low or null neutralizing capacity [3,18]. The characterization of these nonneutralizing anti-MPER antibodies may provide further insights in the mechanisms and molecular determinants of neutralization. For this reason, we aimed to characterize the diverse MPER responses in HIV-1 infected individuals. To this end, we developed small gp41-derived proteins that properly exposed the MPER epitopes recognized by 2F5 and 4E10 BnAbs on the surface of HEK-293T cells. By using cell lines stably transfected with these proteins, we characterized plasma samples from untreated HIV-1 infected individuals. We could detect anti-MPER antibodies in most of these individuals. Furthermore, we found that MPER-specific responses were elicited in the context of a global response against the envelope, which suggest that there is no specific constraint in the elicitation of anti-MPER antibodies. Further characterization of the MPER-specific neutralizing activity showed that anti-MPER responses were highly heterogeneous in terms of neutralization and specific epitope recognition.

## Results

### Generation and characterization of gp41-derived proteins

**Figure 1:**
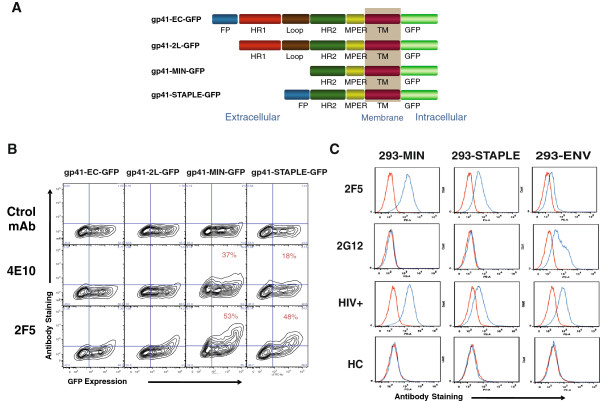
**Characterization of gp41-derived proteins.** Panel **A**. Different gp41-derived proteins used in this study are depicted. The different regions of gp41 are depicted in blue (fusion peptide), red (helicoidal region 1, HR1), brown (disulfide loop), green (HR2), yellow (membrane proximal external region, MPER) and purple (Transmembrane region, TM). The GFP fused to the C-terminal sequence is also depicted in light green. Panel **B**. Flow cytometry analysis of MPER exposure on the surface of transfected cells. 293T cells transiently transfected with the constructions shown in panel A were analyzed for cell surface MPER exposure. Plots of GFP expression and binding of control, 4E10 and 2F5 antibodies are shown. Panel **C**. 293T cells stably expressing the MIN (left panels) or STAPLE (middle panels) constructions were selected and the binding profile of different antibodies was compared with a 293T cell line stably expressing a full-length HIV-1 envelope construct (right panels). Antibodies tested were the anti-MPER mAb 2F5, the anti-gp120 glycan shield mAb 2G12 and plasma samples from HIV-1 infected or uninfected individuals.

We designed a series of proteins containing the MPER of gp41 by generating deletion mutants of gp41 (Figure 1A). Starting from a complete gp41 sequence devoid of the cytoplasmic tail (GP41-EC), we sequentially removed the fusion peptide to generate the GP41-2 L (2 helicoidal regions and loop) protein, the HR1 and the loop region to generate the GP41-MIN protein. Finally, we fused the fusion peptide to the MIN protein to limit HR2 flexibility and to putatively increase the association of the protein to the membrane (GP41-STAPLE construct, Figure 1A). All proteins were cloned in pcDNA3.1 expression vectors fused with a GFP sequence at the C-terminal end and transiently transfected in 293T cells to assess MPER exposure on the surface of transfected cells. As shown in Figure 1B, all proteins were similarly expressed as assessed by the intensity of GFP expression, although the proper exposure of MPER epitopes on the cell surface differed among constructs. The binding of two different anti-MPER antibodies (4E10 and 2F5) to the GP41-EC protein was hardly detectable, and the removal of the fusion peptide had little effect on cell surface MPER exposure, that remained only detectable at low level using the 2F5 antibody. Conversely, removal of the loop and the HR1 region greatly increased MPER exposure that become readily detectable by 4E10 and 2F5 in GP41-MIN transfected cells. Addition of the gp41 fusion peptide at the N-terminal end failed to increase cell surface expression of MPER, rather a decrease was observed for the binding of the 4E10 antibody (Figure 1B).We selected GP41-MIN and GP41-STAPLE constructs to determine the level of anti-MPER antibodies in HIV-1 infected individuals, and generated 293T cell lines stably expressing these proteins. For comparative purposes, a 293T cell line stably expressing the full-length HIV-1 envelope (gp160 protein, isolate NL4.3) was also selected. 293T cells expressing GP41-MIN and GP41-STAPLE showed higher level of cell-surface MPER exposure than cells expressing full-length Env as assessed by 2F5 staining. The low 2F5 signal in the latter cell line was not due to low full-length Env expression, since a strong positive signal was obtained after staining with the 2G12 anti-gp120 antibody (Figure 1C). Plasma from an HIV-1 infected individual showed reactivity against all cells, while background levels of antibody binding were detected when plasma from an uninfected individual was used (Figure 1C).

### Analysis of anti-gp41 responses in HIV-1 infected individuals

**Figure 2:**
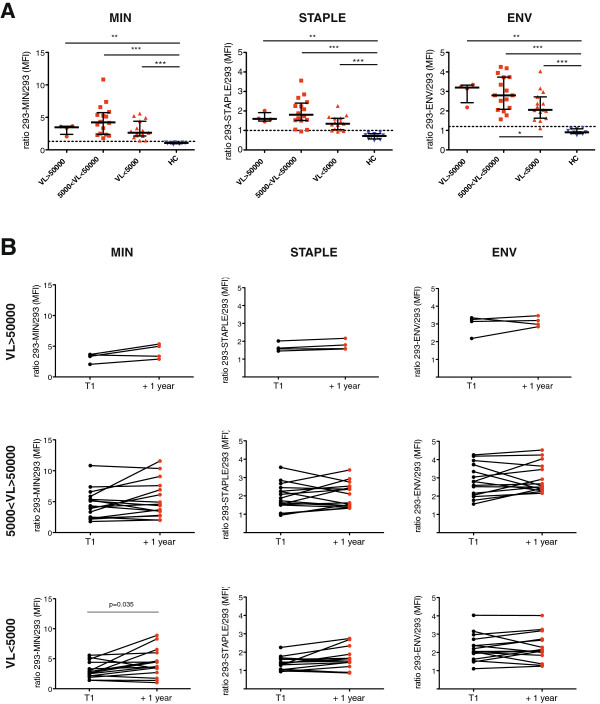
**Identification of anti-MPER antibodies in HIV-1 infected individuals.** Panel **A**. The presence of antibodies recognizing the MIN, STAPLE or full-length HIV-1 envelope was tested using the 293T cells stably expressing these proteins. The upper plots show the ratio of mean fluorescence intensity (MFI) of plasma IgG bound to 293–MIN, 293-STAPLE or 293-ENV (full-length) and control 293 cells. Plasma samples from HIV-1 infected individuals were classified according to plasma viral load (VL > 50000, 50000 < VL < 5000 and VL < 5000). Plasma samples from uninfected individuals (HC) were tested as control. Dotted lines show the positivity cutoff calculated as Mean + 2xSD of uninfected plasma samples. Panel **B**. The longitudinal evolution of MIN, STAPPLE and full-length HIV-1 envelope recognition by plasma samples from HIV-1 infected individuals is shown for the different VL groups defined in Panel **A**. Time points are separated at least one year. All figures show the ratio of MFI between cells stably expressing MIN (left) STAPLE (middle) or full-length envelope (ENV, right) and 293T control cells. Significant p values are shown.

**Figure 3:**
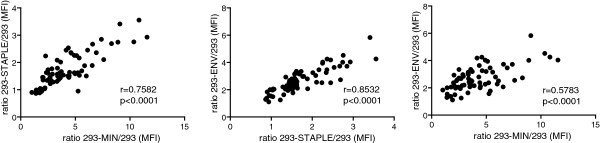
**Antibodies against Gp41-MIN and Gp41-STAPLE are elicited in the context of a wide anti-Env response.** Spearman´s correlation analysis of the signals obtained between 293-MIN and 293-STAPLE cell lines (left panel) and between both cell lines and 293-ENV cell line (middle and right panels, respectively). Figures show the correlation coefficient (r) and p values (p).

Stably transfected cell lines characterized in Figure 1C were used to analyze the binding of 35 plasma samples obtained from viremic untreated HIV-1 infected individuals. Recognition of MIN, STAPLE and full-length Env was assessed by calculation of the ratio of MFI from each plasma obtained in the different cell lines and the MFI obtained using a control untransfected 293T cell line (Figure 2A). Background staining was defined as MEAN + 2xSD of values obtained using 10 plasma samples from uninfected individuals and showed that 94% of samples yield positive signals against GP41-MIN protein, 85% against GP41-STAPLE and 97% against the full-length Env. No major differences among HIV-1 infected individuals were found when samples were classified according to VL, only a lower global anti-Env response was noticed in the group of patient showing VL < 5000 copies/ml (Figure 2A). The stability of humoral responses was assessed using longitudinal samples separated at least one year (Figure 2B) that showed a general conservation of specific responses; only a significant increase was observed for anti-gp41-MIN antibodies in the lowest VL group (Figure 2B), probably associated to a significant increase in VL (data not shown).Furthermore, a strong correlation was observed between the recognition of GP41-MIN and GP41-STAPLE when all samples were analyzed (Figure 3) emphasizing the similarities between both proteins tested. Interestingly, the amount of antibodies bound to both gp41-derived proteins strongly correlated with the total anti-Env response (Figure 3), suggesting that anti-gp41 responses are generated in the context of a potent general anti-Env response.

### Mapping anti-gp41 responses

**Figure 4:**
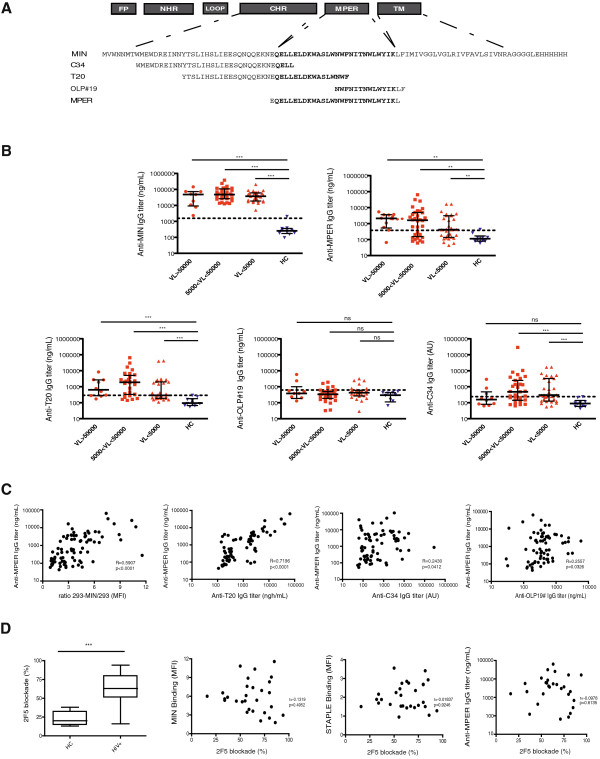
**Mapping anti-gp41 responses.** Panel **A**. Schematic representation of the antigens used for our fine mapping of anti-gp41 responses. Amino acid sequences of a recombinant full-length MIN protein and peptides C34 (gp41 aa 628–661), T20 (gp41 aa 638–673), OLP#19 (gp41 aa 671–684) and MPER (gp41 aa 659–683) are displayed. Panel **B**. Specific IgG titers for the recognition of MIN, MPER, T20, OLP#19 and C34 peptides by plasma samples. Titers are indicated in equivalents of 2F5 in ng/mL for MIN, T-20 and MPER or 4E10 equivalents in ng/ml for OLP#19. For C34 Arbitrary Units (AU) relative to one highly positive plasma sample used as standard are indicated. Panel **C**. Spearman´s correlation between standard anti-MPER ELISA assay and specific IgG signal displayed by 293-MIN cell line stained by plasma samples from HIV-1 infected individuals, anti-T20 ELISA titers, anti-C34 ELISA titers and anti-OLP#19 ELISA titers. Panel **D**. Plasma samples from HIV-1 infected individuals and healthy controls (HC) were tested in a competition assay by using the 293-MIN cell line and a fluorescently-labeled 2F5 antibody. The percentage of blockade of 2F5 binding is shown for both groups of samples. Correlations between 2F5 blockade and specific recognition of 293-MIN, 293-STAPLE and anti-MPER ELISA titers by plasma samples are shown. 2F5 competition assays and fine gp41-peptide mapping confirms the presence of anti-MPER antibodies in plasma from HIV-1 infected individuals. In panel **B** and **D**, ***denotes p < 0,001. In panels **C** and **D** the correlation coefficient (r) and p values (p) are shown.

To determine the peptidic regions recognized by plasma samples and to evaluate the potential differences in recognition of soluble and membrane bound forms of our proteins we performed a series of ELISA assays using a purified full-length MIN protein, the C34, T20, MPER and OLP#19 peptides covering respectively the 628–661, 638–673, 659–683 and 671–684 residues of gp160 (HXB2 numbering, Figure 4A). All plasma samples from HIV-1 infected individuals recognized the soluble form of MIN protein with titers above the cutoff defined by uninfected individuals (Figure 4B). However, the recognition of the MPER peptide yielded positive titers for 66% of plasma samples (Figure 4B). A similar percentage of samples recognized the T20 peptide, which contains the 2F5 core epitope but lacks the 4E10 binding motif (Figure 4B), while a lower percentage of samples (55%) yielded positive titers for C34 peptide binding (Figure 4B) and only 17% of samples recognized the OLP#19 peptide encompassing the 4E10 epitope. Furthermore, a strong positive correlation was found between anti-MPER responses and both the recognition of cell surface expressed MIN protein and anti-T20 titers (Figure 4C), while a poor correlation was observed between anti-MPER titers and either anti-C34 antibodies or anti-4E10 epitope antibodies (Figure 4C). Altogether these data suggest that a robust response against the 2F5 epitope is generated in HIV infected individuals.Therefore, we assayed the functional binding of plasma antibodies to MPER in a competition assay using 293T cells expressing the gp41-MIN protein and labeled 2F5 antibody. As expected, plasma from HIV-1 infected individuals induced a significant blockade of the 2F5 epitope compared to background levels induced by control uninfected samples (Figure 4D). However, the extent of inhibition was not correlated to the titers of anti-MPER antibodies measured by different methods: direct binding to MIN or STAPLE proteins or MPER peptide ELISA (Figure 4D), suggesting heterogeneity in functional binding to the targeted epitopes.

### Neutralization capacity of plasma samples

**Figure 5:**
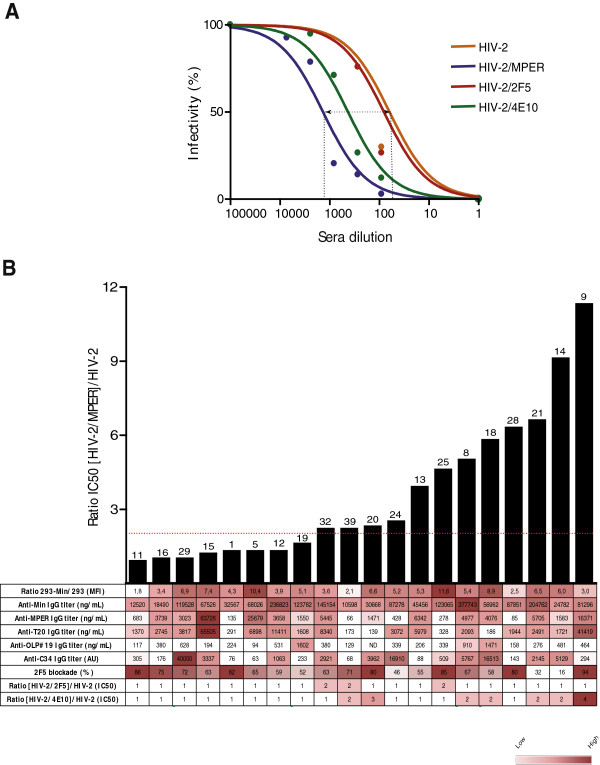
**MPER-like neutralization capacity of selected plasma samples shows diverse antibody specificities.** Panel **A**. Example of the neutralization profile of one plasma sample against a collection of chimeric HIV-2 viruses engrafted with the whole MPER region or the 2F5/4E10 epitopes. Specific neutralization capacity was calculated as the ratio of IC50 between engrafted viruses and wild type HIV-2, corresponding with the curve shift relative to that for wild type HIV-2. Panel **B**. Bar graph shows the level of specific MPER-like neutralization, expressed as described in panel **A**. Numbers on the top of bars indicate patient code. The table displays the values of the different parameters evaluated in the study for each tested plasma sample. Color code is indicated in the lower right corner.

The potency of neutralization of different HIV-1 isolates (NL4.3, BaL, AC10 and SVBP16) was evaluated for plasma samples in TZM-bl cells. A positive correlation was found between the levels of MIN recognition and the neutralization titers for all isolates (data not shown). However, this observation is probably related to the strong correlation between anti-MIN antibodies and global anti-Env responses that may mediate neutralization. Therefore to ascertain the specific neutralizing capacity of anti-MPER antibodies detected in plasma samples, we tested plasma samples against a collection of chimeric HIV-2 viruses engrafted with different MPER sequences [19]. IC-50 values were calculated for the wild type (wt) HIV-2, HIV-2 containing the full MPER sequence (aa 661–684), the 2F5 epitope (aa 661–670) or the 4E10 epitope (aa 671–684). The increase in IC-50 between the wt and the different engrafted viruses was assumed to be the specific contribution of MPER or specific regions to neutralization (Figure 5A). Using this approach, only a percentage of plasma samples showed specific neutralization against the full MPER sequence. Neutralizing activity was hardly detected when using HIV-2 viruses engrafted with shorter epitopes of either 2F5 or 4E10 antibodies. A direct comparison of neutralizing plasma with those lacking neutralization capacity showed an unexpected similarity in ELISA, or flow cytometry parameters evaluated to quantify MPER recognition (Figure 5B). Indeed, several plasma samples exhibited high titers of anti-MPER antibodies in the absence of measurable neutralization capacity (see samples 15, 5 and 12 in Figure 5B), while several plasma samples showed an inverse behavior, with neutralizing capacity in the absence of high anti-MPER titers (samples 25 or 28). Furthermore, the longitudinal analysis of several plasma samples confirmed a robust reproducibility in the different parameters measured, indicating that the very diverse profiles in gp41 humoral responses are stable overtime (see Additional file 1). In summary, these data suggest that both neutralizing and nonneutralizing responses are generated against the MPER epitope and that standard or new epitope binding measurements hardly identify neutralizing activity of polyclonal plasma samples.

## Discussion

The MPER of gp41 is an attractive vaccine candidate that exposes linear peptides as target of broadly neutralizing antibodies. However, the particular localization of the MPER in the HIV-1 envelope glycoprotein trimer may represent a limiting factor for neutralizing activity. Indeed, this sequence is partly inserted in the viral or cellular membrane due to its amphiphilic properties [7], and is located in the base of the inverted pyramid formed by the envelope trimer [20]. However, monoclonal antibodies against the MPER have been shown to exert similar protective effects than anti-gp120 antibodies in non human primate models [5]. Therefore the elicitation of neutralizing anti-MPER responses by several candidate immunogens is still a major issue in HIV vaccine research, although it has been unsuccessful to date [21-24].

All these failed attempts may be explained by the requirement of hydrophobic residues in the CDRH3 loop of anti-MPER antibodies to allow them to access the hydrophobic environment of the targeted sequence [21]. This is the case of the different bnAbs isolated [7,15]. Furthermore, the hydrophobic CDRH3 regions recognize lipids [7,15] and at least 2F5 and 4E10 bnAbs are also cross reactive with human proteins, thus suggesting that tolerance may limit anti-MPER responses [25,26]. All these limitations seem to favor the diversion of humoral immune responses towards other gp41 regions, in particular the external loop, which has been described as an immunodominant nonneutralizing region [27].

To evaluate the impact of the latter limitations in the generation of anti-MPER antibodies, we analyzed the responses against the MPER elicited by natural infection. Responses were quantified by using miniproteins devoid of the immunodominant regions of gp41 but containing the HR2 sequence adjacent to the MPER. Maintaining this region was necessary since removal of the HR2 sequence reduced the binding of 2F5 to the cell surface expressed miniproteins (data not shown, Carrillo et al. in preparation). Unexpectedly, most HIV-1 infected individuals showed specific recognition of two different miniproteins displaying the HR2 and the MPER regions of gp41. In fact, both proteins only differ on the flexibility of the HR2 sequence, being this region free in the MIN protein, while conformationally constrained by the potential interaction of the fusion peptide with the membrane in the STAPLE protein. Thus, the strong association between the recognition of these two proteins suggested that anti-MPER antibodies were responsible for binding. This observation was further confirmed by classical ELISA assays using full length MIN protein, and C34, T20 and MPER peptides; the latter peptides share the N-terminal MPER sequence and showed strong positive correlation in ELISA data. Consistently, a peptide spanning the C-terminal moiety of MPER, OLP#19, showed much lower reactivity. These data reinforced the notion that HIV-1 infected individuals develop anti-MPER antibodies, mainly against the 2F5 epitope, that are stable overtime. Furthermore, another relevant observation is that strong anti-MPER responses occur in patients with strong anti-Envelope responses, suggesting that no specific requirements are needed for such a responses, and that the general immunocompetence, defined by the suboptimal function of CD4 and B cell compartments [28], may be the main limiting factor in the elicitation of strong humoral responses in natural HIV-1 infection. Finally, our analysis also searched for immune correlates of viral control; however, no correlation of viral load was observed with any measured parameter, either related to MPER-specific or general anti-Env responses. This is consistent with previous data showing a lack of correlation between neutralizing activity and virological control and progression to AIDS [29,30].

While the wide presence of anti-MPER responses could be good news for the development of MPER based immunogens, our data point to a more negative aspect, the strong functional heterogeneity of the anti-MPER antibodies detected in HIV-1 infected individuals. We show that plasma samples show divergent neutralizing capacity or ability to block 2F5 binding and that both activities are unrelated to the level of anti-MPER antibodies measured by flow cytometry or ELISA. Several reasons may explain these paradoxical results. The existence of nonneutralizing anti-MPER antibodies, that may compete for the 2F5 binding epitope, has been reported in mice and rhesus macaques immunized with MPER-containing proteins [18,31]. These antibodies seem to be also generated in the context of natural HIV-1 infection and may provide positive results in flow/ELISA assays that detect MPER binding, while failing to induce detectable neutralization. Consistently, a wide analysis of gp41 responses showed that all monoclonal antibodies elicited in humans against the cluster II of gp41 lacked neutralizing activity [32]. Similarly, llama immunization with liposomal formulations of gp41 miniproteins is able to induce neutralizing antibodies despite the absence of neutralizing activity observed in plasma samples [21], suggesting that nonneutralizing antibodies may not only divert neutralizing immune responses but also block the binding of neutralizing antibodies elicited, as shown for gp120 C1 antibodies [33]. However, the ability of nonneutralizing antibodies to bind MPER may allow them to interfere with HIV-1 replication by alternate mechanisms, such as Antibody Dependent Cellular Cytotoxicity (ADCC) that seems to be a protective factor in the RV144 study [34] and has been described for neutralizing anti-MPER antibodies [35]. Our data also provide evidence for a completely opposite setting, the presence of neutralizing antibodies that poorly bind peptidic sequences (this is the case of samples 25 and 28 in Figure 5B and Additional file 1). In these cases, flow cytometry approaches yield higher positivity, suggesting the involvement of lipid membranes in binding to the target epitopes and therefore a benefit of flow cytometry methods to detect these antibodies.

## Conclusions

Given the great complexity of the immunogenicity of the MPER, it seems necessary to better understand the natural humoral response to this region. For instance, further characterization of new monoclonal antibodies isolated from HIV-1 infected individuals displaying high titers of anti-MPER antibodies with neutralizing and nonneutralizing activity will be beneficial for the definition of the mechanisms and the structural requirements involved in the elicitation of broadly neutralizing antibodies. In this regard, the role of nonneutralizing antibodies should be defined. These data will provide an improved framework for the design of novel MPER-based immunogens that directs the humoral response towards neutralizing activities.

## Methods

### Samples

We selected plasma samples from untreated HIV-1 infected individuals according to the following criteria: HAART naïve individuals with VL > 50 copies/ml, with at least two plasma samples available separated by one year. A total of 35 individuals fulfilled selection criteria and were classified in three groups according to the VL in the first time-point analyzed: Group 1: VL > 50000, Group 2: 50000 > VL > 5000 and Group 3: VL < 5000 copies/mL. Blood from 10 uninfected healthy donors were collected by venipuncture. Plasma was prepared by blood centrifugation for 10 minutes at 3000 × g.

### Construction of Gp41 derivatives

The pHXB2 plasmid containing a full HIV-1 sequence and the peGFP plasmid containing an eGFP coding sequence were used to amplify gp41 and GFP sequences. The primers were designed according to published sequences, and were as follows (5´-3´): i) for the fusion peptide region GP41-EC-f **CACC**ATGGCAGTGGGAATAGGAGCTATG and FP-r *TACCGTCAGC*GTCATTGAGGCTG; ii) for the HR1 region GP41-2 L-f **CACC**ATGCAGGCCAGACAATTATTGTCTG; iii) for the HR2 region GP41-MAX-f **CACC**ATGATTTGGAATAACATGACCTGG and HR-2 f-2 *GCTGACGGTA*ATTTGGAATCACACGACCTGG; iv) for the transmembrane region GP41-r *GCCACCGCCACC*TAGCTCTATTCACTATAGA and v) for the intracellular region GP41-MAX-r *GCCACCGCCACC*TAGCAAAATCCTTTCCA. The bold text indicates overhang sequences for directional cloning. The italic text indicates overlapping sequences for the generation of fused proteins. All amplification reactions were performed using Amplitaq DNA polymerase (Applied Biosystems) in a 2720 Thermal cycler (Applied Biosystems). For each construct, an additional plasmid containing the green fluorescent protein (GFP) coding sequence fused to gp41 in C terminal was constructed. The final PCR products were cloned into a pcDNA vector using the pcDNA 3.1 Directional TOPO® Expression Kit (Invitrogen). The positive clones were selected in ampicillin plates, recovered, screened by PCR, sequenced and then tested for functional expression. A previously reported pcDNA3.1 plasmid coding for the NL4.3 envelope gene was used to express the full-length envelope [36].

### Transient and stable expression in 293T cells

Human embryonic kidney HEK-293T cells (ATCC Accession No. CRL-11268) were cultured in DMEM medium (Invitrogen) supplemented with 10% fetal calf serum (Invitrogen). One day before transfection, the cells were detached using versene (Invitrogen), washed in supplemented DMEM and split in six well plates at a density of 400000 cells/well. For transfection, each well was transfected with 2 μg of one of the plasmids coding for gp41constructs described herein using the CalPhos Mammalian Transfection Kit (Clontech). Transient expression was assayed 24–48 hours after transfection.

Stable expression of gp41 proteins was assayed by flow cytometry after culturing transfected cells in supplemented DMEM containing 1 mg/ml of the selection antibiotic G418 (Invitrogen). Transient and stable expressions were assayed by determining cell surface expression and total levels of gp41 proteins in cell extracts. In addition, molecular weight, western blot analysis, MPER integrity, antibody recognition and residual fusogenic activity assays were also performed.

293T cells were stained with the anti-gp41 2F5 or 4E10 monoclonal antibodies (Polymun Scientific) at a concentration of 4 μg/ml for 30 minutes at room temperature. After washing, bound antibodies were revealed using a PE-labeled Goat-anti-human IgG (Jackson ImmunoResearch) and analyzed in a LSR II flow cytometer (Becton Dickinson). The level of expression was determined as the % of positive cells or the Mean Fluorescence Intensity (MFI). Mock transfected HEK-293T cells were used as negative controls. The ratio of MFI observed for samples and negative controls was measured as a surrogate parameter of MPER exposure.

### Peptides and proteins

HIV-1 IIIB C34 and T20 peptides were obtained through the NIH AIDS Reagent Program (Division of AIDS, NIAID). A 28-mer MPER peptide (EQELLELDKWASLWNWFNITNWLWYIKL) was ordered to ThermoFisher Scientific. The OLP#19 peptide covering the C-terminal part of MPER was kindly provided by C. Brander (IrsiCaixa, Spain). MIN sequence was cloned in a pET-21d(+) expression vector (Novagen) and produced by *E. coli* BL21 DE3 strain (Invitrogen). Inclusion bodies were prepared from 1 L of bacterial culture and solubilised using 8 M urea. Highly pure protein was obtained through niquel-based Immobilized Metal Affinity Chromatography (GE Healthcare) and gel filtration using a Sephacryl S-100 HR column (GE Healthcare).

### Enzyme Linked Immunosorbent Assays

Peptides C34, T20, OLP#19, MPER and recombinant MIN protein were coated in 96-well Maxisorp Nunc-immuno plates (Fisher Scientific). After blocking, plates were incubated with 100 ul of previously diluted plasma samples overnight at 4°C. Plates were then washed and 100 ul of a Horseradish Peroxidase (HRP)-conjugated F(ab)2 Goat antihuman IgG (Fc specific) (Jackson Immunoresearch) were dispensed for one hour at room temperature. Plates were developed with 100 ul of O-Phenylenediamine dihydrochloride (OPD) substrate (Sigma-Aldrich) and stopped with 100 ul of 4 N H_2_SO_4_. Optical density was measured at 492 nm for specific signal and at 620 nm for background.

### 2F5 competition assay

The 2F5 antibody was labeled with the DyLight 649 Microscale Antibody Labeling Kit (Pierce) and titrated in 293T cells expressing MIN protein. Competition of plasma samples with labeled 2F5 was performed by preincubating 293-MIN cells with 1/10 dilutions of plasma for 15 minutes at room temperature, and then with 0.5 ug/ml of 2F5 for 30 min. Cells were washed in PBS, fixed in FA 1% in PBS and analyzed by flow cytometry.

### Viruses and neutralization assays

HIV-2 chimeras were made in the context of the full-length p7312A HIV-2 molecular clone (GenBank accession number L36874). Expression vectors for the wild type HIV-2 (p7312A) and HIV-2 chimeras containing the HIV-1 gp41 Membrane Proximal External Region (p7312A-C1), the 2F5 (p7312A-C3) or 4E10 epitopes (p7312A-C4), were kindly provided by G.M Shawn (University of Pennsylvania) [19]. Pseudoviruses were generated by transfection of plasmids in 293T cells. After 24 hours post-transfection, supernatants were harvested, filtered at 0.45 micron and viral stocks frozen at -80°C.

HIV-1 isolates NL4.3, BaL, AC10 and SVP16 were generated as pseudoviruses using Env expression plasmids and the pSG3 vector as described [37]. Cell-free virus neutralization by plasma samples was tested by a standard TZM-bl based assay [38]. Briefly, in a 96-well culture plate, 100 ul of previously diluted plasma samples were preincubated with 50 ul of pseudovirus stock, using 200 TCID50, at 37°C, one hour. Then, 100 ul containing 10,000 TZM-bl luciferase-reporter target cells per well were added. Plates were cultured at 37°C and 5% CO2 for 48 hours. 2F5, 4E10 and IgGb12 (Polymun Scientific), and anti-CD4 clone SK3 (BD Biociences) were used as controls. Plasma samples were inactivated (56°C, 30 minutes) prior to the assay and threefold serial dilutions were tested, from 1/60 to 1/4960. TZM-bl reporter cells were treated with dextran (Sigma Aldrich) to enhance infectivity. Luciferase substrate, Britelite Plus (Perkin-Elmer) was used for the read out.

### Statistical analysis

Variables were expressed as the median (interquartile range) and compared using Mann–Whitney test. Spearman’s correlation coefficient was calculated to assess the association between variables. Non-linear fit of neutralization data were calculated using normalized values fitted to an one-site inhibition curve with fixed Hill slope [39]. All statistical analyses and non-linear fitting were performed using the GraphPad Prism v5.0 software. Positivity cutoffs for ELISA and flow cytometry assays were calculated using the MEAN + 2xSD of values obtained using HIV-1 seronegative individuals. Bonferroni correction has been calculated for multiple comparisons.

## Competing interests

MIN and STAPLE proteins are protected by the WO/2012/055985 patent.

## Authors’ contributions

EG, MC and JC developed the different gp41-derivatives. LMM-A, MLRC and SM performed most of the experimental work (ELISA, competition and neutralization assays). BC and JB selected patient samples and designed the study. All authors read and approved the final manuscript.

## Supplementary Material

Additional file 1 Analysis of stability overtime of different markers for anti-MPER humoral response. Click here for file

## References

[B1] Muster T Steindl F Purtscher M Trkola A Klima A Himmler G Rüker F Katinger H A conserved neutralizing epitope on gp41 of human immunodeficiency virus type 1 J Virol 1993 67 6642 6647 7692082 10.1128/jvi.67.11.6642-6647.1993 PMC238102

[B2] Stiegler G Kunert R Purtscher M Wolbank S Voglauer R Steindl F Katinger H A potent cross-clade neutralizing human monoclonal antibody against a novel epitope on gp41 of human immunodeficiency virus type 1 AIDS Res Hum Retroviruses 2001 17 1757 1765 10.1089/08892220152741450 11788027

[B3] Zwick MB Labrijn AF Wang M Spenlehauer C Saphire EO Binley JM Moore JP Stiegler G Katinger H Burton DR Parren PW Broadly neutralizing antibodies targeted to the membrane-proximal external region of human immunodeficiency virus type 1 glycoprotein gp41 J Virol 2001 75 10892 10905 10.1128/JVI.75.22.10892-10905.2001 11602729 PMC114669

[B4] Huang J Ofek G Laub L Louder MK Doria-Rose NA Longo NS Imamichi H Bailer RT Chakrabarti B Sharma SK Alam SM Wang T Yang Y Zhang B Migueles SA Wyatt R Haynes BF Kwong PD Mascola JR Connors M Broad and potent neutralization of HIV-1 by a gp41-specific human antibody Nature 2012 491 406 412 10.1038/nature11544 23151583 PMC4854285

[B5] Hessell AJ Rakasz EG Tehrani DM Huber M Weisgrau KL Landucci G Forthal DN Koff WC Poignard P Watkins DI Burton DR Broadly neutralizing monoclonal antibodies 2F5 and 4E10 directed against the human immunodeficiency virus type 1 gp41 membrane-proximal external region protect against mucosal challenge by simian-human immunodeficiency virus SHIVBa-L J Virol 2010 84 1302 1313 10.1128/JVI.01272-09 19906907 PMC2812338

[B6] McCoy LE Weiss RA Neutralizing antibodies to HIV-1 induced by immunization J Exp Med 2013 210 209 223 10.1084/jem.20121827 23401570 PMC3570100

[B7] Montero M van Houten NE Wang X Scott JK The membrane-proximal external region of the human immunodeficiency virus type 1 envelope: dominant site of antibody neutralization and target for vaccine design Microbiol Mol Biol Rev 2008 72 54 84 10.1128/MMBR.00020-07 18322034 PMC2268283

[B8] Denner J Towards an AIDS vaccine: the transmembrane envelope protein as target for broadly neutralizing antibodies Hum Vaccin 2011 7 4 9 10.4161/hv.7.0.14555 21266839

[B9] Kim M Song L Moon J Sun Z-YJ Bershteyn A Hanson M Cain D Goka S Kelsoe G Wagner G Irvine D Reinherz EL Immunogenicity of membrane-bound HIV-1 gp41 membrane-proximal external region (MPER) segments is dominated by residue accessibility and modulated by stereochemistry J Biol Chem 2013 288 31888 31901 10.1074/jbc.M113.494609 24047898 PMC3814781

[B10] Montero M Gulzar N Klaric K-A Donald JE Lepik C Wu S Tsai S Julien J-P Hessell AJ Wang S Lu S Burton DR Pai EF Degrado WF Scott JK Neutralizing epitopes in the MPER of HIV-1 gp41 are influenced by the transmembrane domain and the plasma membrane J Virol 2012 86 2930 2941 10.1128/JVI.06349-11 22238313 PMC3302331

[B11] Haynes BF Fleming J St Clair EW Katinger H Stiegler G Kunert R Robinson J Scearce RM Plonk K Staats HF Ortel TL Liao H-X Alam SM Cardiolipin polyspecific autoreactivity in two broadly neutralizing HIV-1 antibodies Science 2005 308 1906 1908 10.1126/science.1111781 15860590

[B12] Alam SM McAdams M Boren D Rak M Scearce RM Gao F Camacho ZT Gewirth D Kelsoe G Chen P Haynes BF The role of antibody polyspecificity and lipid reactivity in binding of broadly neutralizing anti-HIV-1 envelope human monoclonal antibodies 2F5 and 4E10 to glycoprotein 41 membrane proximal envelope epitopes J Immunol 2007 178 4424 4435 10.4049/jimmunol.178.7.4424 17372000 PMC2262928

[B13] Finton KAK Larimore K Larman HB Friend D Correnti C Rupert PB Elledge SJ Greenberg PD Strong RK Autoreactivity and exceptional CDR plasticity (but not unusual polyspecificity) hinder elicitation of the anti-HIV antibody 4E10 PLoS Pathog 2013 9 e1003639 10.1371/journal.ppat.1003639 24086134 PMC3784475

[B14] Reardon PN Sage H Dennison SM Martin JW Donald BR Alam SM Haynes BF Spicer LD Structure of an HIV-1-neutralizing antibody target, the lipid-bound gp41 envelope membrane proximal region trimer Proc Natl Acad Sci U S A 2014 111 1391 1396 10.1073/pnas.1309842111 24474763 PMC3910588

[B15] Chen J Frey G Peng H Rits-Volloch S Garrity J Seaman MS Chen B Mechanism of HIV-1 neutralization by antibodies targeting a membrane-proximal region of gp41 J Virol 2014 88 1249 1258 10.1128/JVI.02664-13 24227838 PMC3911647

[B16] Sun Z-YJ OKJ Kim M Yu J Brusic V Song L Qiao Z Wang J-H Wagner G Reinherz EL HIV-1 broadly neutralizing antibody extracts its epitope from a kinked gp41 ectodomain region on the viral membrane Immunity 2008 28 52 63 10.1016/j.immuni.2007.11.018 18191596

[B17] Gach JS Leaman DP Zwick MB Targeting HIV-1 gp41 in close proximity to the membrane using antibody and other molecules Curr Top Med Chem 2011 11 2997 3021 10.2174/156802611798808505 22044228

[B18] Alam SM Scearce RM Parks RJ Plonk K Plonk SG Sutherland LL Gorny MK Zolla-Pazner S Vanleeuwen S Moody MA Xia S-M Montefiori DC Tomaras GD Weinhold KJ Karim SA Hicks CB Liao H-X Robinson J Shaw GM Haynes BF Human immunodeficiency virus type 1 gp41 antibodies that mask membrane proximal region epitopes: antibody binding kinetics, induction, and potential for regulation in acute infection J Virol 2008 82 115 125 10.1128/JVI.00927-07 17942537 PMC2224348

[B19] Dhillon AK Donners H Pantophlet R Johnson WE Decker JM Shaw GM Lee F-H Richman DD Doms RW Vanham G Burton DR Dissecting the neutralizing antibody specificities of broadly neutralizing sera from human immunodeficiency virus type 1-infected donors J Virol 2007 81 6548 6562 10.1128/JVI.02749-06 17409160 PMC1900098

[B20] Mao Y Wang L Gu C Herschhorn A Xiang S-H Haim H Yang X Sodroski J Subunit organization of the membrane-bound HIV-1 envelope glycoprotein trimer Nat Struct Mol Biol 2012 19 893 899 10.1038/nsmb.2351 22864288 PMC3443289

[B21] Lutje Hulsik D Liu Y-Y Strokappe NM Battella S El Khattabi M McCoy LE Sabin C Hinz A Hock M Macheboeuf P Bonvin AMJJ Langedijk JPM Davis D Forsman Quigley A Aasa-Chapman MMI Seaman MS Ramos A Poignard P Favier A Simorre J-P Weiss RA Verrips CT Weissenhorn W Rutten L A gp41 MPER-specific Llama VHH requires a hydrophobic CDR3 for neutralization but not for antigen recognition PLoS Pathog 2013 9 e1003202 10.1371/journal.ppat.1003202 23505368 PMC3591319

[B22] Venditto VJ Watson DS Motion M Montefiori D Szoka FC Rational design of membrane proximal external region lipopeptides containing chemical modifications for HIV-1 vaccination Clin Vaccine Immunol 2013 20 39 45 10.1128/CVI.00615-12 23114698 PMC3535774

[B23] Strasz N Morozov VA Kreutzberger J Keller M Eschricht M Denner J Immunization with hybrid proteins containing the membrane proximal external region of HIV-1 AIDS Res Hum Retroviruses 2014 30 498 508 doi:10.1089/AID.2013.0191. Epub 2014 Feb 7. 10.1089/aid.2013.0191 24392780 PMC4010167

[B24] Ye L Wen Z Dong K Wang X Bu Z Zhang H Compans RW Yang C Induction of HIV neutralizing antibodies against the MPER of the HIV envelope protein by HA/gp41 chimeric protein-based DNA and VLP vaccines PLoS One 2011 6 e14813 10.1371/journal.pone.0014813 21625584 PMC3098228

[B25] Chen Y Zhang J Hwang K-K Bouton-Verville H Xia S-M Newman A Ouyang Y-B Haynes BF Verkoczy L Common tolerance mechanisms, but distinct cross-reactivities associated with gp41 and lipids, limit production of HIV-1 broad neutralizing antibodies 2F5 and 4E10 J Immunol 2013 191 1260 1275 10.4049/jimmunol.1300770 23825311 PMC3725147

[B26] Verkoczy L Diaz M Holl TM Ouyang Y-B Bouton-Verville H Alam SM Liao H-X Kelsoe G Haynes BF Autoreactivity in an HIV-1 broadly reactive neutralizing antibody variable region heavy chain induces immunologic tolerance Proc Natl Acad Sci U S A 2010 107 181 186 10.1073/pnas.0912914107 20018688 PMC2806760

[B27] Enshell-Seijffers D Smelyanski L Vardinon N Yust I Gershoni JM Dissection of the humoral immune response toward an immunodominant epitope of HIV: a model for the analysis of antibody diversity in HIV + individuals FASEB J 2001 15 2112 2120 10.1096/fj.00-0898com 11641237

[B28] Moir S Fauci AS B cells in HIV infection and disease Nature Reviews Immunology 2009 9 235 245 10.1038/nri2524 19319142 PMC2779527

[B29] Doria-Rose NA Klein RM Daniels MG O'Dell S Nason M Lapedes A Bhattacharya T Migueles SA Wyatt RT Korber BT Mascola JR Connors M Breadth of human immunodeficiency virus-specific neutralizing activity in sera: clustering analysis and association with clinical variables J Virol 2010 84 1631 1636 10.1128/JVI.01482-09 19923174 PMC2812355

[B30] Euler Z van Gils MJ Bunnik EM Phung P Schweighardt B Wrin T Schuitemaker H Cross-reactive neutralizing humoral immunity does not protect from HIV type 1 disease progression J Infect Dis 2010 201 1045 1053 10.1086/651144 20170371

[B31] Dennison SM Anasti K Scearce RM Sutherland L Parks R Xia S-M Liao H-X Gorny MK Zolla-Pazner S Haynes BF Alam SM Nonneutralizing HIV-1 gp41 envelope cluster II human monoclonal antibodies show polyreactivity for binding to phospholipids and protein autoantigens J Virol 2011 85 1340 1347 10.1128/JVI.01680-10 21106741 PMC3020517

[B32] Pietzsch J Scheid JF Mouquet H Seaman MS Broder CC Nussenzweig MC Anti-gp41 antibodies cloned from HIV-infected patients with broadly neutralizing serologic activity J Virol 2010 84 5032 5042 10.1128/JVI.00154-10 20219932 PMC2863839

[B33] Tomaras GD Ferrari G Shen X Alam SM Liao H-X Pollara J Bonsignori M Moody MA Fong Y Chen X Poling B Nicholson CO Zhang R Lu X Parks R Kaewkungwal J Nitayaphan S Pitisuttithum P Rerks-Ngarm S Gilbert PB Kim JH Michael NL Montefiori DC Haynes BF Vaccine-induced plasma IgA specific for the C1 region of the HIV-1 envelope blocks binding and effector function of IgG Proc Natl Acad Sci U S A 2013 110 9019 9024 10.1073/pnas.1301456110 23661056 PMC3670311

[B34] Bonsignori M Pollara J Moody MA Alpert MD Chen X Hwang K-K Gilbert PB Huang Y Gurley TC Kozink DM Marshall DJ Whitesides JF Tsao C-Y Kaewkungwal J Nitayaphan S Pitisuttithum P Rerks-Ngarm S Kim JH Michael NL Tomaras GD Montefiori DC Lewis GK DeVico A Evans DT Ferrari G Liao H-X Haynes BF Antibody-dependent cellular cytotoxicity-mediating antibodies from an HIV-1 vaccine efficacy trial target multiple epitopes and preferentially use the VH1 gene family J Virol 2012 86 11521 11532 10.1128/JVI.01023-12 22896626 PMC3486290

[B35] Tudor D Bomsel M The broadly neutralizing HIV-1 IgG 2F5 elicits gp41-specific antibody-dependent cell cytotoxicity in a FcγRI-dependent manner AIDS 2011 25 751 759 10.1097/QAD.0b013e32834507bd 21330910

[B36] Curriu M Fausther-Bovendo H Pernas M Massanella M Carrillo J Cabrera C López-Galíndez C Clotet B Debré P Vieillard V Blanco J Viremic HIV infected individuals with high CD4 T cells and functional envelope proteins show anti-gp41 antibodies with unique specificity and function PLoS One 2012 7 e30330 10.1371/journal.pone.0030330 22312424 PMC3270019

[B37] Sánchez-Palomino S Massanella M Carrillo J García A García F González N Merino A Alcamí J Bofill M Yuste E Gatell JM Clotet B Blanco J A cell-to-cell HIV transfer assay identifies humoral responses with broad neutralization activity Vaccine 2011 29 5250 5259 10.1016/j.vaccine.2011.05.016 21609746

[B38] Li M Gao F Mascola JR Stamatatos L Polonis VR Koutsoukos M Voss G Goepfert P Gilbert P Greene KM Bilska M Kothe DL Salazar-Gonzalez JF Wei X Decker JM Hahn BH Montefiori DC Human immunodeficiency virus type 1 env clones from acute and early subtype B infections for standardized assessments of vaccine-elicited neutralizing antibodies J Virol 2005 79 10108 10125 10.1128/JVI.79.16.10108-10125.2005 16051804 PMC1182643

[B39] Blanco J Canela EI Mallol J Lluís C Franco R Characterization of adenosine receptors in brush-border membranes from pig kidney Br J Pharmacol 1992 107 671 678 10.1111/j.1476-5381.1992.tb14505.x 1335333 PMC1907783

