# Supplementary material for: Anti-MPER antibodies with heterogeneous neutralization capacity are detectable in most untreated HIV-1 infected individuals
Source: Retrovirology. 2014 Jun 7;11:44. doi: 10.1186/1742-4690-11-44 (PMC4067070; doi:10.1186/1742-4690-11-44)
Supplement: Additional file 1 — Analysis of stability overtime of different markers for anti-MPER humoral response. [file 1742-4690-11-44-S1.pdf]

Additional file 1.

Molinos-Albert & Carrillo et al.

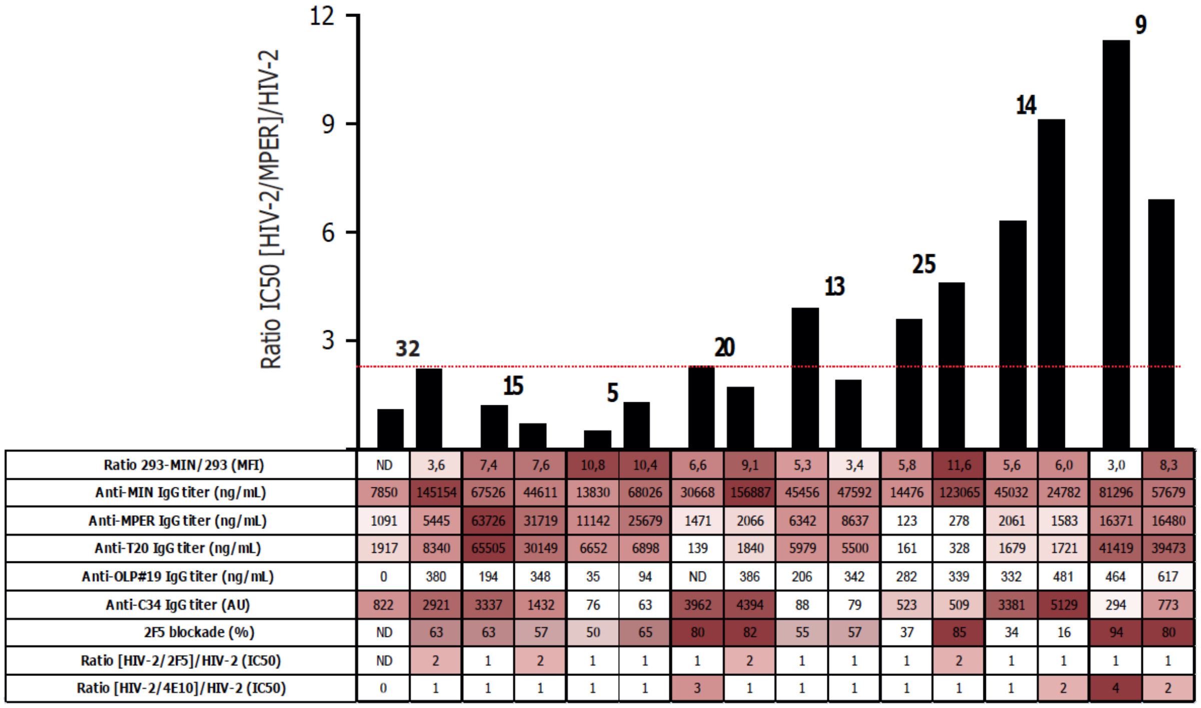

Ratio 293-MIN/293 (MFI)	ND	3,6	7,4	7,6	10,8	10,4	6,6	9,1	5,3	3,4	5,8	11,6	5,6	6,0	3,0	8,3
Anti-MIN IgG titer (ng/mL)	7850	145154	67526	44611	13830	68026	30668	156887	45456	47592	14476	123065	45032	24782	81296	57679
Anti-MPER IgG titer (ng/mL)	1091	5445	63726	31719	11142	25679	1471	2066	6342	8637	123	278	2061	1583	16371	16480
Anti-T20 IgG titer (ng/mL)	1917	8340	65505	30149	6652	6898	139	1840	5979	5500	161	328	1679	1721	41419	39473
Anti-OLP#19 IgG titer (ng/mL)	0	380	194	348	35	94	ND	386	206	342	282	339	332	481	464	617
Anti-C34 IgG titer (AU)	822	2921	3337	1432	76	63	3962	4394	88	79	523	509	3381	5129	294	773
2F5 blockade (%)	ND	63	63	57	50	65	80	82	55	57	37	85	34	16	94	80
Ratio [HIV-2/2F5]/HIV-2 (IC50)	ND	2	1	2	1	1	1	2	1	1	1	2	1	1	1	1
Ratio [HIV-2/4E10]/HIV-2 (IC50)	0	1	1	1	1	1	3	1	1	1	1	1	1	2	4	2

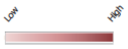

Longitudinal analysis of MPER-like neutralization capacity of selected plasma samples. Bar graph show the level of specific MPER-like neutralization, expressed as described in Figure 5 for longitudinal analysis of plasma samples. Numbers on the top of paired bars indicate patient code. The table displays the values of the different parameters evaluated in the study for each plasma tested. Color code indicated in the lower right corner and corresponds to that used in Figure 5.
